# Supplementary material for: Inflammatory Cells Can Alter the Levels of H3K9ac and γH2AX in Dysplastic Cells and Favor Tumor Phenotype
Source: J Pers Med. 2023 Apr 13;13(4):662. doi: 10.3390/jpm13040662 (PMC10141380; doi:10.3390/jpm13040662)
Supplement: Supplementary file 1 [file jpm-13-00662-s001.zip › jpm-2313735-Supplementary Materials.pdf]

Table S1. Primers used in RT-qPCR assays.

Target	Forward (5’→3’)	Reverse (5’→3’)
GAPDH	GAAGGTGAAGGTCGCAGTC	GAAGATGGTGATGGATTTC
E-cadherin	ACAGCCCCGCCTTATGATT	TCGGAACCGCTTCCTTCA
N-cadherin	GTGCATGAAGGACAGCCTCT	CCACCTTAAAATCTGCAGGC
Vimentin	GGCTCGTCACCTTCGTGAAT	TCAATGTCAAGGGCCATCTTAA

Table S2. Antibodies against cell cycle proteins and epithelial-mesenchymal transition markers used in the western blot assay.

Antibody	Clone	Company	kDa	Dilution
β-actin	ACTB	ABclonal	43	1:2000
H3K9ac	C5B11	Cell Signaling	17	1:500
γH2AX	6L16	Sigma-Aldrich	15	1:1000
E-Cadherin	24E10	Cell Signaling	135	1:1000
N-Cadherin	D4R1H	Cell Signaling	140	1:500
Vimentin	D21H3	Cell Signaling	57	1:1000
$p16^{INK4}$	G175-405	BD Pharmingen	16	1:100
$p21^{WAF1/Cip1}$	C-19	Santa Cruz	21	1:200
$p27^{Kip1}$	57/Kip1/pp27	BD Pharmingen	27	1:200
CDK2	55/CDK2	BD Pharmingen	33	1:1000
CDK4	C-22	Santa Cruz	34	1:5000
CDK6	C-21	Santa Cruz	36	1:1000
Cyclin D1	Polyclonal	ABclonal	36	1:2000
Cyclin E	E-4	Santa Cruz	53	1:1000
